# Effects of mild obesity on outcomes in Japanese patients with COVID-19: a nationwide consortium to investigate COVID-19 host genetics

**DOI:** 10.1038/s41387-022-00217-z

**Published:** 2022-08-09

**Authors:** Ho Lee, Shotaro Chubachi, Ho Namkoong, Hiromu Tanaka, Shiro Otake, Kensuke Nakagawara, Atsuho Morita, Takahiro Fukushima, Mayuko Watase, Tatsuya Kusumoto, Katsunori Masaki, Hirofumi Kamata, Makoto Ishii, Naoki Hasegawa, Norihiro Harada, Tetsuya Ueda, Soichiro Ueda, Takashi Ishiguro, Ken Arimura, Fukuki Saito, Takashi Yoshiyama, Yasushi Nakano, Yoshikazu Mutoh, Yusuke Suzuki, Koji Murakami, Yukinori Okada, Ryuji Koike, Yuko Kitagawa, Akinori Kimura, Seiya Imoto, Satoru Miyano, Seishi Ogawa, Takanori Kanai, Koichi Fukunaga, The Japan COVID-19 Task Force

**Affiliations:** 1 Division of Pulmonary Medicine, Department of Medicine, Keio University School of Medicine, Shinjuku-ku, Tokyo Japan; 2 Department of Infectious Diseases, Keio University School of Medicine, Shinjuku-ku, Tokyo Japan; 3 Department of Respiratory Medicine, Juntendo University Faculty of Medicine and Graduate School of Medicine, Tokyo, Japan; 4 Department of Respiratory Medicine, Osaka Saiseikai Nakatsu Hospital, Osaka, Japan; 5 JCHO (Japan Community Health Care Organization) Saitama Medical Center, Internal Medicine, Saitama, Japan; 6 Department of Respiratory Medicine, Saitama Cardiovascular and Respiratory Center, Kumagaya, Japan; 7 Department of Respiratory Medicine, Tokyo Women’s Medical University, Tokyo, Japan; 8 Department of Emergency and Critical Care Medicine, Kansai Medical University General Medical Center, Moriguchi, Japan; 9 Respiratory Diseases Center, Fukujuji Hospital, Tokyo, Japan; 10 Department of Internal Medicine, Kawasaki Municipal Ida Hospital, Kawasaki, Japan; 11 Department of Infectious Diseases, Tosei General Hospital, Seto, Japan; 12 Department of Respiratory Medicine, Kitasato University Kitasato Institute Hospital, Tokyo, Japan; 13 Department of Respiratory Medicine, Tohoku University Graduate School of Medicine, Sendai, Japan; 14 Department of Statistical Genetics, Osaka University Graduate School of Medicine, Suita, Japan; 15 Medical Innovation Promotion Center, Tokyo Medical and Dental University, Tokyo, Japan; 16 Department of Surgery, Keio University School of Medicine, Tokyo, Japan; 17 Institute of Research, Tokyo Medical and Dental University, Tokyo, Japan; 18 Division of Health Medical Intelligence, Human Genome Center, the Institute of Medical Science, the University of Tokyo, Tokyo, Japan; 19 M&D Data Science Center, Tokyo Medical and Dental University, Tokyo, Japan; 20 Department of Pathology and Tumor Biology, Kyoto University, Kyoto, Japan; 21 Division of Gastroenterology and Hepatology, Department of Medicine, Keio University School of Medicine, Tokyo, Japan

**Keywords:** Obesity, Risk factors

## Abstract

**Background:**

Obesity is reported to be a risk factor for severe disease in patients with coronavirus disease 2019 (COVID-19). However, there are no specific reports on the risk of severe disease according to body mass index (BMI) in Japan. Thus, this study aimed to investigate the effect of obesity stratified by BMI on the severity of COVID-19 in the general Japanese population.

**Methods:**

From February 2020 to May 2021, 1 837 patients aged ≥18 years were enrolled in the Japan COVID-19 Task Force. Patients with known BMI and disease severity were analyzed. Severity was defined as critical if the patient was treated in the intensive care unit, required invasive mechanical ventilation, or died.

**Results:**

Class 1 obesity (25.0 ≤ BMI < 30.0 kg/m^2^), class 2 obesity (30.0 ≤ BMI < 35.0 kg/m^2^), and class 3 or 4 obesity (BMI ≥ 35 kg/m^2^) were present in 29%, 8%, and 3% of the cases, respectively. Multiple logistic regression analysis with known risk factors for critical illness indicated that class 2 obesity was an independent risk factor for oxygenation (adjusted odds ratio, 4.75) and critical cases (adjusted odds ratio, 1.81). Class 1 obesity and class 3 or 4 obesity were independent risk factors for oxygen administration (adjusted odds ratios 2.01 and 3.12, respectively), but not for critical cases. However, no differences in the mortality rates were observed between the BMI classes (*P* = 0.5104).

**Conclusion:**

Obesity is a risk factor for respiratory failure in Japanese patients with COVID-19, regardless of the degree of obesity. However, it may not cause severe COVID-19 in a dose–response relationship with BMI. COVID-19 patients with mild obesity may benefit from aggressive intensive care.

## Introduction

Coronavirus disease 2019 (COVID-19) is an infectious disease caused by the severe acute respiratory syndrome coronavirus 2 [1]. It is widespread in many countries and progresses from mild viral illness to hypoxia, multiple organ failure, acute respiratory distress syndrome, and death [2]. Although several factors that contribute to the development of severe COVID-19 have been identified, such as increasing age, male sex, geographic region, and multiple chronic comorbidities, obesity is emerging as a significant risk factor, especially in industrialized countries [3–5]. In the United States of America, severe obesity with a body mass index (BMI) ≥ 35 kg/m^2^ has been reported to be a risk factor for invasive mechanical ventilation (IMV), intensive care unit (ICU) admission, and hospital death [4, 5].

The prevalence of obesity in the Japanese population is lower than that in Westerners [6]. In the United States of America, 40% of the population have obesity (BMI ≥ 30 kg/m^2^), and 9% of the population has a BMI ≥ 40 kg/m^2^ [7]. Meanwhile, the percentage of people with obesity in Japan is approximately 4.5%, and the percentage of people with a BMI > 35 kg/m^2^ is approximately 0.9% [8]. Recently, a genome-wide association study has identified host genetic factors that contribute to the risk of developing severe COVID-19 with respiratory failure [9, 10]. We have conducted a nationwide multicenter consortium to overcome the COVID-19 pandemic in Japan (https://www.covid19-taskforce.jp/en/home/). Previously, we reported the association between obesity-related genes and COVID-19 severity using a Mendelian randomization analysis [11]. Thus, obesity may be a significant comorbidity in Japanese patients with COVID-19. However, the relationship between obesity and COVID-19 severity in the Japanese population, which differs greatly from that of Westerners (white and black) in terms of the number of infections and deaths and the percentage of obesity, has not yet been clarified. Therefore, we hypothesized that the frequency and impact of obesity on disease severity might be different from those reported in Westerners. This study aimed to investigate the effect of obesity stratified by BMI on the severity of COVID-19 in the general Japanese population.

## Subjects and methods

### Study design and settings

**Fig. 1:**
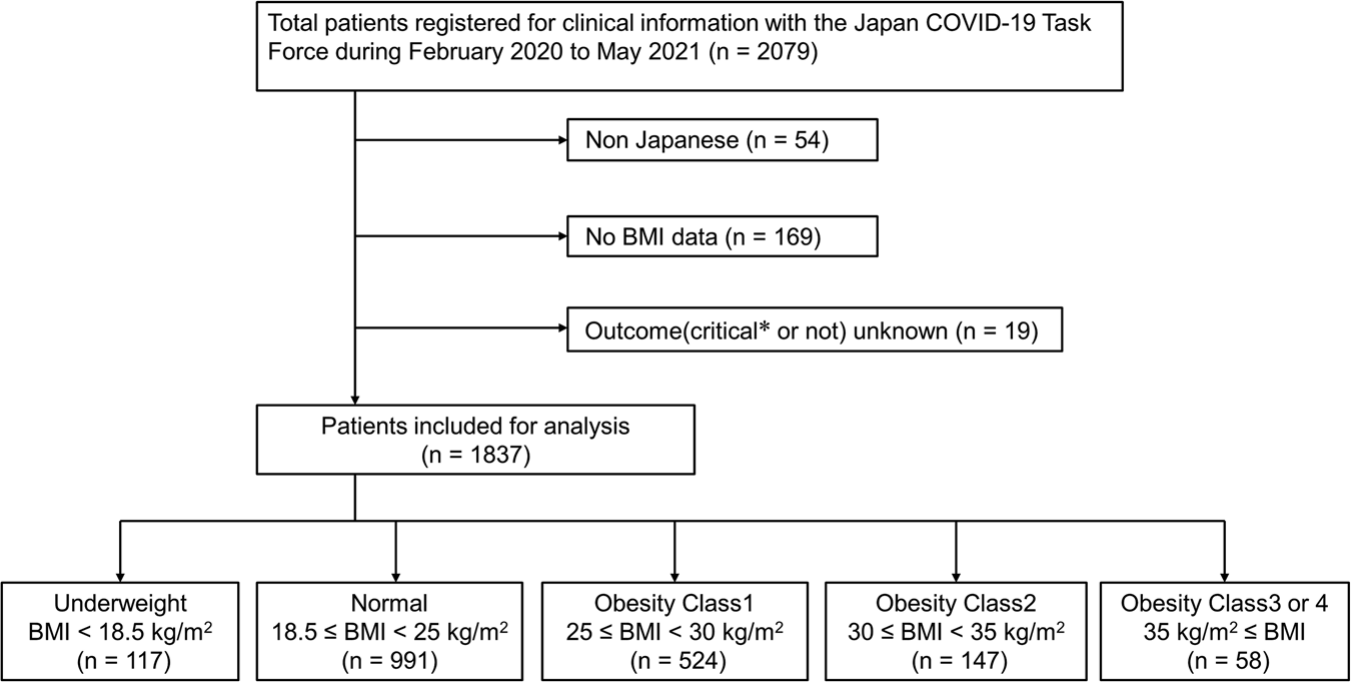
Flowchart describing the patient selection. All consecutive patients with COVID-19 aged ≥18 years who were hospitalized during the study period and recruited through the Japan COVID-19 Task Force between February 2020 and May 2021 were included. After excluding 242 patients, 1837 patients were enrolled in this study. *A critical case was defined as receiving treatment in the intensive care unit, using invasive mechanical ventilation, or hospital death.

All cases affected by COVID-19 were recruited through the Japan COVID-19 Task Force [11]. From February 2020 to May 2021, data from consecutive patients aged ≥18 years who were diagnosed with COVID-19 using severe acute respiratory syndrome coronavirus 2 polymerase chain reaction test results at one of more than 100 affiliated hospitals and who agreed to cooperate in the study were registered in an electronic case record form by the study subspecialist at the affiliated research institute and were analyzed in this retrospective cohort study. The inclusion criteria were: (i) non-Japanese patients and (ii) patients with incomplete medical records, such as inability to obtain BMI and critical outcome information. Of the 2 079 patients who met the exclusion criteria, we excluded 54 non-Japanese patients and 188 patients without BMI and outcome information. Thus, 1837 patients were included in the analysis (Fig. 1).

This study was approved by the Ethics Committee of Keio University School of Medicine (ID: 20200061), and written or oral informed consent was obtained. The study was performed in accordance with the ethical standards laid down in the 1964 Declaration of Helsinki and its later amendments.

### Data collection

Actual measurement values of height and weight at admission were obtained from physicians, and BMI was calculated. The following data were extracted from the electronic case record form: age, sex, clinical symptoms and signs, laboratory and radiographic findings on admission, comorbidities, and disease severity (ICU entry, using IMV, and survival status). All laboratory tests were performed according to the clinical care needs of the patients. Symptoms and signs were included at the time of referral and admission and at the time of hospitalization. Laboratory and radiographic results were collected within 48 h of the initial visit or admission. The collected data were reviewed by a team of respiratory clinicians. If core data were missing, the clinician was contacted to collect the data. Missing data in the patient background were noted as unknown.

### Outcomes and statistics

The primary exposure in all analyses was BMI. BMI was calculated using the height and weight recorded during hospitalization. BMI categories were defined using the following Ministry of Health, Labour and Welfare, Japan criteria: underweight (BMI < 18.5 kg/m^2^), normal weight (18.5 ≤ BMI < 25.0 kg/m^2^), class 1 obesity (25.0 ≤BMI < 30.0 kg/m^2^), class 2 obesity (30.0 ≤BMI < 35.0 kg/m^2^), and class 3 or 4 obesity (BMI ≥ 35 kg/m^2^). The primary outcome was critical illness, defined as treatment in the ICU, using IMV, or death [5, 12]. Continuous and categorical variables are presented as mean ± standard deviation (SD) or number (proportion), respectively. Data were compared among the five groups using an analysis of variance and the χ^2^ test as appropriate. Additionally, among patients with BMI ≥ 25 kg/m^2^, we compared clinical information between the critical and non-critical groups. Student’s *t* test and the χ^2^ test were used to compare the two groups.

We performed univariate and multivariate logistic regression analyses to evaluate the relationship between BMI and COVID-19 severity: oxygen administration, ICU treatment, IMV use, and critical illness. Multivariate logistic regression analyses were performed on factors reported as risk factors for severe disease in previous studies and factors selected in previous studies (BMI groups, age, sex, and presence of comorbidities: hypertension, diabetes, prior cardiovascular disease, and chronic kidney disease) [13–17]. Odds ratios (ORs) and adjusted odds ratios (aORs) with 95% CIs were used in the comparison. In all outcome analyses, we predefined the group without obesity (underweight or normal: BMI < 25 kg/m^2^) as the reference group. All P-values were two-tailed, and statistical significance was set at *P* < 0.05. All data were analyzed using the JMP 16 program (SAS Institute Japan Ltd., Tokyo, Japan).

## Results

### Comparison of baseline characteristics by obesity classes

The baseline characteristics of the patients are shown in Table 1. The proportions of each BMI category were underweight (6%), normal weight (54%), class 1 obesity (29%), class 2 obesity (8%), and class 3 or 4 obesity (3%). High BMI classes were associated with younger age (*P* < 0.0001). Additionally, patients of high BMI classes were more likely to be male (*P* < 0.0001) and had more comorbidities such as hypertension (*P* < 0.0001), diabetes (*P* < 0.0001), hyperuricemia (*P* < 0.0001), and chronic liver disease (*P* = 0.023). On admission, patients with a higher BMI had a higher prevalence of fever (*P* = 0.0068), cough (*P* = 0.0001), shortness of breath (*P* < 0.0001), and sense of fatigue (*P* = 0.0008).

**Table 1:** Baseline characteristics of patients by body mass index category.

Characteristics	Underweight (*n* = 117)	Normal (*n* = 991)	Class 1 obesity (*n* = 524)	Class 2 obesity (*n* = 147)	Class 3 or 4 obesity (*n* = 58)	*P* value
Weight	43.1 (±6.2)	60.0 (±9.0)	75.1 (±9.4)	89.0 (±10.9)	109.8 (±20.7)	**<0.0001**
Age, years	63.5 (±22.9)	59.2 (±18.6)	58.5 (±14.9)	54.9 (±3.3)	47.8 (±14.1)	**<0.0001**
Male	48 (41.0)	628 (63.4)	399 (76.2)	109 (74.2)	42 (72.4)	**<0.0001**
*Symptoms*						
Confusion	7 (6.1)	38 (3.9)	16 (3.1)	6 (4.1)	1 (1.8)	0.5427
Fever	86 (74.5)	756 (76.8)	422 (81.5)	122 (83.6)	48 (87.3)	**0.0409**
Cough	52 (44.4)	569 (57.9)	325 (62.7)	103 (71.5)	35 (62.5)	**0.0001**
Sputum	28 (24.1)	233 (23.9)	120 (23.1)	45 (31.3)	13 (22.8)	0.3628
Sore throat	22 (19.1)	236 (24.3)	115 (22.3)	43 (29.5)	10 (17.9)	0.2109
Shortness of breath	23 (20.4)	277 (28.9)	201 (39.0)	60 (41.4)	22 (40.0)	**<0.0001**
Abdominal pain	1 (0.9)	24 (2.5)	19 (3.7)	7 (4.8)	1 (1.7)	0.2336
Diarrhea	13 (11.4)	159 (16.3)	96 (18.6)	29 (19.7)	12 (20.7)	0.2737
Nausea or vomiting	11 (9.6)	94 (9.7)	40 (7.7)	10 (6.9)	4 (7.0)	0.6119
Sense of fatigue	41 (35.7)	462 (47.4)	267 (51.3)	89 (61.0)	30 (52.6)	**0.0008**
*Admission vital signs*						
Temperature	37.3 (±0.9)	37.2 (±0.9)	37.3 (±1.0)	37.5 (±1.0)	37.4 (±1.0)	**0.0068**
Systolic blood pressure, mmHg	122.8 (±22.0)	128.1 (±20.5)	130.5 (±18.6)	130.6 (±20.2)	132.9 (±18.9)	**0.001**
Diastolic blood pressure, mmHg	74.8 (±14.2)	79.0 (±13.0)	82.1 (±13.1)	84.9 (±14.1)	85.6 (±14.9)	**<0.0001**
Heartrate beat/min	86.3 (±17.3)	85.6 (±16.2)	87.8 (±15.9)	93.0 (±16.7)	96.3 (±21.0)	**<0.0001**
Oxygenation saturation < 94%	24 (20.5)	274 (28.0)	171 (33.0)	63 (43.8)	18 (31.0)	**0.0002**
*Comorbidities*						
Hypertension	31 (30.0)	292 (30.0)	216 (41.6)	87 (59.2)	27 (48.2)	**<0.0001**
Diabetes	15 (12.9)	183 (18.6)	146 (28.0)	57 (39.0)	28 (48.3)	**<0.0001**
Prior cardiovascular disease	17 (14.7)	91 (9.3)	53 (10.2)	15 (10.3)	1 (1.7)	0.0978
Cancer	13 (11.4)	74 (7.6)	29 (5.6)	4 (2.8)	3 (5.2)	0.0444
Chronic obstructive pulmonary disease	5 (4.4)	55 (5.6)	20 (3.9)	5 (3.5)	0 (0)	0.1895
Asthma	7 (6.1)	54 (5.6)	442 (8.2)	11 (7.7)	8 (13.8)	0.0717
Hyperuricemia	6 (5.2)	68 (6.9)	85 (16.4)	27 (18.5)	10 (17.5)	**<0.0001**
Chronic liver disease	1 (0.9)	33 (3.5)	16 (3.2)	8 (5.8)	7 (12.3)	**0.0023**
Chronic kidney disease	9 (8.0)	69 (7.3)	48 (9.6)	10 (7.4)	4 (7.0)	0.644
Smoking, current or former	27 (25.5)	424 (46.2)	247 (50.5)	65 (47.5)	24 (43.6)	**0.0002**

Data are presented as the mean ± SD or n(%).

Body mass index (BMI) was categorized as underweight (BMI < 18.5 kg/m^2^), normal (18.5 ≤ BMI < 25.0 kg/m^2^), class 1 obesity (25.0 ≤ BMI < 30.0 kg/m^2^), class 2 obesity (30.0 ≤ BMI < 35.0 kg/m^2^), or class 3 or 4 obesity (BMI ≥ 35.0 kg/m^2^).

### Comparison of laboratory and imaging findings by obesity classes

The laboratory parameters and imaging findings of the patients are shown in Table 2. Patients with higher BMI had higher levels of hemoglobin (*P* < 0.0001), aspartate aminotransferase (*P* < 0.0001), alanine aminotransferase (*P* < 0.0001), γ-glutamyl transpeptidase (*P* < 0.0001), lactate dehydrogenase (*P* < 0.0001), uric acid (*P* < 0.0001), complement C3 (*P* < 0.0001), serum ferritin (*P* < 0.0001), triglyceride (*P* < 0.0001), Krebs von den Lungen-6 (*P* = 0.0001), and hemoglobin A1c (*P* < 0.0001). Such patients also had a higher frequency of bilateral ground glass opacity on chest radiography (*P* < 0.0001), computed tomography (*P* = 0.018), and consolidation on chest radiography (*P* < 0.0001) and computed tomography (*P* = 0.0004).

**Table 2:** Laboratory and imaging findings on presentation by body mass index category.

Characteristics	Underweight (*n* = 117)	Normal (*n* = 991)	Class 1 obesity (*n* = 524)	Class 2 obesity (*n* = 147)	Class 3 or 4 obesity (*n* = 58)	P value
*Laboratory parameters*						
White blood cell/μl	5393 (±2799)	5794 (±3138)	5950 (±2834)	6070 (±3207)	6109 (±2279)	0.3077
Neutrophil percentage, %	69 (±15)	70 (±13)	70 (±13)	68 (±13)	69 (±13)	0.3426
Lymphocytes percentage, %	22 (±12)	21 (±14)	22 (±10)	23 (±10)	23 (±11)	0.7006
Hemoglobin, g/l	12.8 (±1.9)	13.8 (±1.9)	14.5 (±1.7)	15.0 (±1.8)	15.2 (±1.7)	**<0.0001**
Platelet count ×10^4^/μl	18.9 (±5.6)	20.1 (±7.4)	19.8 (±8.6)	19.2 (±6.9)	20.3 (±6.9)	0.359
Albumin, g/dl	3.6 (±0.8)	3.7 (±0.6)	3.7 (±0.6)	3.8 (±0.6)	3.8 (±0.5)	0.1624
Total bilirubin, mg/dl	0.6 (±0.4)	0.7 (±0.4)	0.7 (±0.3)	0.7 (±0.3)	0.7 (±0.3)	0.9255
AST, U/l	33 (±43)	37 (±34)	41 (±29)	48 (±29)	68 (±105)	**<0.0001**
ALT, U/l	21 (±21)	32 (±31)	43 (±38)	55 (±42)	73 (±62)	**<0.0001**
γ-GTP, U/l	35 (±44)	58 (±80)	78 (±95)	96 (±93)	97 (±73)	**<0.0001**
ALP, U/l	184 (±103)	175 (±124)	175 (±124)	174 (±114)	186 (±91)	0.9149
BUN, mg/dl	19 (±18)	18 (±16)	17 (±11)	17 (±13)	16 (±14)	0.5467
Serum creatinine, mg/dl	1.2 (±2.0)	1.1 (±1.5)	1.1 (±1.2)	1.1 (±1.3)	1.4 (±2.7)	0.5812
LDH, U/l	233 (±94)	267 (±121)	296 (±188)	321 (±158)	322 (±177)	**<0.0001**
Urine acid, mg/dl	4.6 (±2.2)	4.7 (±1.8)	5.0 (±1.6)	5.4 (±2.0)	5.8 (±2.0)	**<0.0001**
Creatinine kinase, U/l	162 (±433)	155 (±573)	169 (±311)	148 (±162)	207 (±365)	0.9178
BNP, pg/ml	182 (±356)	81 (±486)	55 (±260)	27 (±51)	54 (±134)	0.2559
C3, mg/dl	93 (±18)	119 (±26)	128 (±31)	132 (±22)	137 (±24)	**<0.0001**
C4, mg/dl	32 (±11)	36 (±12)	39 (±15)	38 (±10)	40 (±11)	0.2175
Serum ferritin, ng/ml	405 (±703)	496 (±559)	710 (±754)	780 (±732)	692 (±568)	**<0.0001**
Triglyceride, mg/dl	93 (±49)	117 (±71)	141 (±109)	177 (±252)	198 (±71)	**<0.0001**
KL-6, U/ml	296 (±222)	296 (±226)	372 (±437)	406 (±409)	323 (±183)	**0.0001**
HbA1c, %	5.9 (±1.0)	6.2 (±1.1)	6.6 (±1.5)	6.8 (±1.4)	7.3 (±2.1)	**<0.0001**
Fibrinogen, mg/dl	399 (±136)	482 (±156)	510 (±155)	489 (±124)	482 (±123)	**<0.0001**
D-dimer, μg/dl	3.5 (±8.3)	2.5 (±9.4)	2.2 (±7.3)	2.3 (±9.4)	1.3 (±1.2)	0.5169
Procalcitonin, ng/ml	0.48 (±1.89)	0.29 (±1.54)	0.33 (±2.99)	0.15 (±0.35)	0.36 (±1.68)	0.8524
CRP, mg/dl	4.6 (±8.0)	4.7 (±5.9)	5.6 (±6.4)	5.0 (±5.0)	4.8 (±4.5)	0.1431
*Imaging*						
Chest radiography ground glass opacities						**<0.0001**
Unilateral	16 (15.38)	117 (12.70)	59 (11.78)	16 (11.35)	6 (10.71)	
Bilateral	35 (33.65)	483 (52.44)	309 (61.68)	86 (60.99)	35 (62.50)	
Chest radiography consolidation						**0.018**
Unilateral	18 (17.31)	67 (7.35)	42 (8.48)	13 (9.22)	3 (5.36)	
Bilateral	13 (12.50)	207 (22.72)	101 (20.40)	35 (24.82)	10 (17.86)	
Chest computed tomography ground glass opacities						**<0.0001**
Unilateral	17 (17.89)	95 (10.37)	47 (9.57)	12 (8.89)	4 (7.14)	
Bilateral	49 (51.58)	632 (69.00)	374 (76.17)	118 (87.41)	46 (82.14)	
Chest computed tomography consolidation						**0.0004**
Unilateral	18 (19.15)	70 (7.87)	32 (6.71)	8 (6.02)	1 (1.85)	
Bilateral	17 (18.09)	286 (32.13)	165 (34.59)	50 (37.59)	16 (29.63)	

Data are presented in mean ± SD or n(%).

Body mass index (BMI) was categorized as underweight (BMI < 18.5 kg/m^2^), normal (18.5≤ BMI < 25.0 kg/m^2^),

Class 1 obesity (25.0 ≤ BMI < 30.0 kg/m^2^), Class 2 obesity (30.0≤BMI < 35.0 kg/m^2^), or Class 3 or 4 obesity (BMI ≥ 35.0 kg/m^2^).

*AST*  aspartate aminotransferase, *ALT*  alanine aminotransferase, *γ-GTP* γ-glutamyl transpeptidase, *ALP* alkaline phosphatase, *BUN* blood urea nitrogen, *LDH* lactate dehydrogenase, *BNP* brain natriuretic peptide, *C3* complement C3, *C4* complement C4, *KL-6* Krebs von den Lungen-6, *HbA1c* hemoglobin A1c, *CRP* C-reactive protein.

### Association between clinical outcomes and obesity classes

**Fig. 2:**
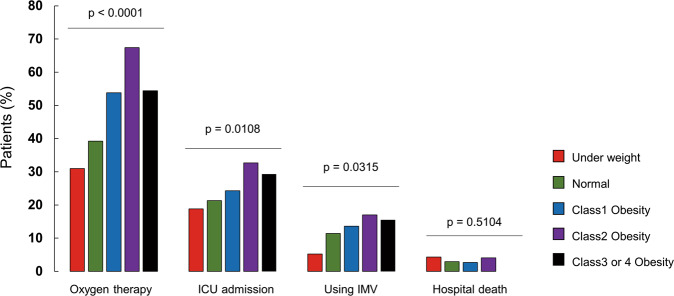
Comparison of COVID-19 severity by BMI. Comparison of outcomes (oxygen therapy, treatment in intensive care unit, use of invasive mechanical ventilation, and hospital death) according to body mass index classes.

**Fig. 3:**
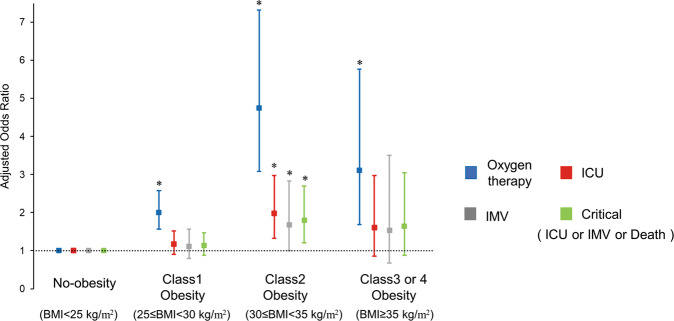
Risk of oxygen therapy, treatment in intensive care unit, use of invasive mechanical ventilation, and critical illness (treatment in intensive care unit or using invasive mechanical ventilation or hospital death) according to body mass index (BMI). Forest plot of adjusted odds ratio and 95% confidence intervals according to BMI category by multivariate logistic regression analyses. Outcomes were adjusted for BMI groups, age, sex, and presence of comorbidities such as hypertension, diabetes, prior cardiovascular disease, and chronic kidney disease.

The outcomes of patients stratified by BMI are shown in Fig. 2. Patients with higher BMI had higher rates of oxygen therapy (*P* < 0.0001), ICU treatment (*P* = 0.0108), and IMV (*P* = 0.0315). In contrast, in-hospital deaths were few and not significantly different (*P* = 0.5104). The results of the univariate and multivariate logistic regression analyses are shown in the supplementary file and Fig. 3. The univariate logistic regression analysis indicated that compared to underweight or normal, patients with class 1 obesity (OR = 1.86 [1.50–2.30]), class 2 obesity (OR = 3.30 [2.28–4.77]), and class 3 or 4 obesity (OR = 1.91 [1.12–3.25]) were at higher risk of requiring oxygen therapy (supplementary file). However, class 2 obesity had the highest OR. Treatment in the ICU was associated with a higher risk of having class 1 (OR = 1.28 [1.00–1.63]) and class 2 obesity (OR = 1.82 [1.25–2.64]) than being without obesity. Using IMV (OR = 1.70 [1.06–2.72]) and critical illness (OR = 1.63 [1.13–2.36]) were at a higher risk than non-obesity only for class 2 obesity. Multivariate logistic regression analysis also showed that patients with class 1 obesity (aOR = 2.01 [1.56–2.57]), class 2 obesity (aOR = 4.75 [3.08–7.32]), and class 3 or 4 obesity (OR = 3.12 [1.68–5.77]) were at higher risk of oxygen therapy than those with no obesity (Fig. 3). Moreover, ICU treatment (aOR = 1.99 [1.32–2.98]), using IMV (aOR = 1.68 [1.00–2.83]), and critical illness (aOR = 1.81 [1.21–2.70]) were at higher risk than non-obesity only for class 2 obesity.

### Comparison of baseline characteristics in the obesity category according to critical illness

The characteristics of patients with higher BMI classes classified as critical and non-critical are shown in Table 3. No significant differences in BMI were observed between critical or non-critical illness group (*P* = 0.174). Age was significantly higher in the critical illness group than in the non-critical illness group (*P* < 0.0001). Additionally, the critical illness group had significantly more comorbidities such as hypertension (*P* = 0.004), diabetes (*P* = 0.0184), chronic obstructive pulmonary disease (*P* = 0.0352), and chronic kidney disease (*P* = 0.0009) than the non-critical illness group. These results indicated that among patients in the high BMI category, older age and comorbidities play a more significant role in COVID-19 severity than among those with higher BMI.

**Table 3:** Baseline characteristics in the obesity category with and without critical illnesses.

Characteristics	No critical illness (*n* = 517)	Critical illness (*n* = 212)	*P* value
Weight	80.3 (±14.7)	81.7 (±15.7)	0.2353
BMI	28.9 (±3.9)	29.4 (±4.7)	0.174
Age, y	55.5 (±15.4)	60.4 (±13.0)	**<0.0001**
Male	382 (73.9)	168 (79.3)	0.1269
*Symptoms*			
Confusion	4 (0.8)	19 (9.1)	**<0.0001**
Fever	423 (83.1)	169 (80.5)	0.4008
Cough	324 (63.7)	139 (66.5)	0.4681
Sputum	133 (26.0)	45 (21.6)	0.2209
Sore throat	120 (23.6)	48 (22.9)	0.8257
Shortness of breath	178 (34.9)	105 (51.0)	**<0.0001**
Abdominal pain	15 (2.9)	12 (5.7)	0.0746
Diarrhea	104 (20.4)	33 (15.6)	0.1389
Nausea or vomiting	39 (7.7)	15 (7.1)	0.8049
Sense of fatigue	272 (53.0)	114 (54.0)	0.8051
*Admission vital signs*			
Temperature	37.4 (±0.9)	37.3 (±1.1)	0.2473
Heartrate beat/min	90.4 (±16.1)	87.2 (±18.1)	**0.0185**
Systolic blood pressure, mmHg	131.7 (±17.7)	128.4 (±21.7)	**0.0375**
Diastolic blood pressure, mmHg	84.1 (±13.2)	80.0 (±13.8)	**0.0002**
Oxygenation saturation < 94%	128 (25.0)	124 (59.6)	**<0.0001**
*Comorbidities*			
Hypertension	217 (42.3)	113 (54.1)	**0.004**
Diabetes	150 (29.2)	81 (38.2)	**0.0184**
Prior cardiovascular disease	43 (8.4)	26 (12.3)	0.0975
Cancer	26 (5.1)	10 (4.8)	0.8562
Chronic obstructive pulmonary disease	13 (2.5)	12 (5.7)	**0.0352**
Asthma	43 (8.5)	18 (8.7)	0.946
Hyperuricemia	78 (15.3)	44 (21.1)	0.0602
Chronic liver disease	22 (4.5)	9 (4.3)	0.9084
Chronic kidney disease	32 (6.6)	30 (14.4)	**0.0009**
Smoking, current or former	243 (50.5)	99 (49.3)	0.7631

Data are presented in mean ± SD or n(%).

## Discussion

To the best of our knowledge, this is the first large-scale study to investigate the prevalence of obesity stratified by BMI and the clinical characteristics of Japanese COVID-19 patients with obesity. In comparison with the population of the Japanese National Nutrition Survey [8], Japanese patients hospitalized for COVID-19 were more likely to have obesity. Moreover, the number of patients with obesity was lower than that reported in Western studies [17–19]. One of the major strengths of this study is the comprehensive assessment of clinical data, including BMI. Only one previous study has reported the impact of obesity in Japanese patients with COVID-19 [20]. However, missing BMI data were supplemented by physicians. Given the large number of cases with detailed clinical data and accurate BMI measured by physicians, we were able to reveal that there is no dose–response relationship between obesity and COVID-19 severity and that mild obesity is important in Japanese people.

In many Western reports, severe obesity has been associated with poor outcomes [21, 22]. However, our study is clinically significant to note that even patients with class 2 obesity had poor outcomes other than death. These patients had high rates of respiratory failure, ICU admission, and IMV use. Patients with class 1 obesity also had poor outcomes on oxygen administration. However, no differences in the mortality rates were observed between the BMI classes, suggesting that patients with class 1 obesity or class 2 obesity might benefit from aggressive intensive care. In the United States and Europe, there are reports of increased severity of illness and mortality, especially in patients with severe obesity [4, 18, 19]. Here, severe obesity had no significant effect on the outcome, although this may be an underestimation due to the small number of patients with severe obesity. Our study revealed that patients with obesity and critical illness had significantly more comorbidities than those with obesity without critical illness. However, no significant difference in BMI was observed between the two groups. These results were consistent with the absence of a dose–response relationship between BMI and clinical outcomes in this study, indicating that obesity is an essential factor contributing to poor COVID-19 outcomes, while critical illness in patients with obesity is a multifaceted condition involving other factors such as comorbidities.

Obesity-related adverse events in COVID-19 may involve both mechanical and inflammatory mechanisms. First, obesity suppresses diaphragmatic movement and limits chest wall mobility, which may adversely affect lung function and cause hypoxemia due to atelectasis and shunting, thereby contributing to worsened breathing during infection [18, 23, 24]. Second, severe acute respiratory syndrome coronavirus 2 cell invasion is mediated by angiotensin-converting enzyme 2 receptor [25]. Individuals with obesity have enhanced expression of angiotensin-converting enzyme 2 receptor in the adipocytes. Thus, the presence of excess adipose tissue may increase the severity of the infection, and obesity and COVID-19 severity may be associated [26, 27]. Third, obesity is associated with chronic inflammation due to increased pro-inflammatory cytokines and leptin by adipocytes and immune cells, including elevated C-reactive protein, interleukin 6, and ferritin [28, 29]. In COVID-19, higher inflammatory biomarkers indicate greater disease severity [12, 30]. However, in the present study, ferritin levels suggested a significant difference in comparison between groups according to BMI, while C-reactive protein levels did not indicate a significant difference. A previous study has also reported no correlation between high BMI and interleukin 6, C-reactive protein, and ferritin levels in patients with COVID-19 [19, 30, 31]. Ultimately, several factors may contribute to the pathophysiology of obesity and COVID-19 severity, and further studies are needed.

This study had several limitations. The first limitation was the selection of patients; only hospitalized patients were included. Since only inpatients were included in the study, the number of patients who became seriously ill increased, and the risk of obesity causing serious illness may have been overestimated. Second, the analysis was based only on the BMI and host factors. In the early stages of the disease, such as the first wave in Japan, steroids were avoided, although corticosteroids were later found to be effective [32]. The number of effective treatments such as remdesivir, baricitinib, and tocilizumab increased [33, 34]. As described above, significant differences were observed between the first wave of treatment and current treatment, which may have affected the outcomes since the present study did not analyze the timing of treatment. Third, the virus strains have mutated in the data collection, and each mutated strain may have different characteristics [35]. Since the mutant strains were not included in the analysis in this study, the results may be different if they were included in the analysis. Fourth, East Asian ethnic groups are characterized by more visceral fat for lower BMI, and visceral fat has been reported to be a major prognostic factor for COVID-19 [36–38]. This study may have underestimated visceral fat in the Japanese population. It would be good if waist circumference or, even better, chest computed tomography-derived visceral fat itself could be used as a parameter, but this was not possible in the present study. Fifth, the number of patients with class 3 or 4 obesity was small in this study, and it is possible that the impact of severe obesity on COVID-19 severity was not accurately assessed. Therefore, further large-scale studies are desirable in the future. Owing to these limitations, further studies are required.

In conclusion, the number of patients with obesity was lower than that reported in Westerners. Patients with higher BMI classes had more comorbidities and a higher prevalence of respiratory and systemic symptoms. All higher BMI classes were associated with oxygen administration. However, obesity may not increase severity in a dose-dependent manner; only class 2 obesity may be associated with critical illness. COVID-19 patients with mild obesity may benefit from aggressive intensive care.

## Supplementary information

all consortium members

Supplemental table

## Data Availability

The data that support the findings of this study are available from the corresponding author upon reasonable request.
